# The Antinociceptive and Anti-Inflammatory Activities of *Aspidosperma tomentosum* (Apocynaceae)

**DOI:** 10.1155/2013/218627

**Published:** 2013-05-28

**Authors:** Anansa Bezerra de Aquino, Luiz Henrique Agra Cavalcante-Silva, Carolina Barbosa Brito da Matta, Willians Antônio do Nascimento Epifânio, Pedro Gregório Vieira Aquino, Antônio Euzébio Goulart Santana, Magna Suzana Alexandre-Moreira, João Xavier de Araújo-Júnior

**Affiliations:** ^1^LaFI—Laboratório de Farmacologia e Imunidade, Instituto de Ciências Biológicas e da Saúde, Universidade Federal de Alagoas, Maceió, AL, Brazil; ^2^Laboratório de Pesquisa em Recursos Naturais, Instituto de Química e Biotecnologia, Universidade Federal de Alagoas, Maceió, AL, Brazil

## Abstract

We investigated the antinociceptive and anti-inflammatory activities of the crude ethanolic extract (CEE), its fractions, and the flavonoid isorhamnetin from *Aspidosperma tomentosum* using models of nociception and inflammation in mice. In the writhing test, the CEE and its fractions (except for soluble phase, CHCl_3_ 100% and EtAcO 100%) at 100 mg/kg p.o. induced antinociceptive activity. Isorhamnetin (100 **μ**mol/kg, p.o.) was also active. In the hot plate test, only the treatment with the fractions Hex : CHCl_3_ 50%, CHCl_3_ 100%, and CHCl_3_ : MeOH 5% (100 mg/kg, p.o.) increased the latency time, reversed by the opioid antagonist naloxone. Fractions that were active in the hot plate test did not show catalepsy condition. It was observed that CEE, all fractions, and isorhamnetin reduced the formalin effects in the neurogenic phase. In the inflammatory phase, only CEE, isorhamnetin, and CHCl_3_ 100% and CHCl_3_ : MeOH 5% fractions were active. CEE and all fractions, except for CHCl_3_ : MeOH 10% fraction, isorhamnetin, and soluble fraction were able to produce an antioedematogenic activity in the ear capsaicin-induced edema test. In the thioglycolate-induced peritonitis, only EtAcO 100% fraction was not active. The results demonstrate that *A. tomentosum* has antinociceptive and anti-inflammatory activities in animal models.

## 1. Introduction 

Inflammation is a nonspecific response of the organism to invasion by a foreign body, such as microorganism. This response is characterized by five signals: (i) redness and heat, resulting from an increase in blood flow; (ii) swelling which is associated with increased vascular permeability; (iii) pain as the consequence of activation and sensitization of primary afferent nerve fibers; and (iv) loss of function [1]. In this regard the study of mechanisms and mediators involved in painful and inflammatory processes has been the subject of numerous studies over the past years [2–4]. 

Although there are several products used for the treatment of these processes, so far, there is no ideal anti-inflammatory or analgesic compound, either by limiting their effectiveness or by the spread of their adverse effects [5]. In this regard it is important to note that many currently available anti-inflammatory drugs may be associated with significant complications, including the following: gastrointestinal hemorrhage, heart attack, and stroke, which represents an important limitation to use of these products [6–8]. For this reason, the search for substances that have potent anti-inflammatory activity and with limited adverse effects is still stimulating the scientific community. Thus the natural compounds, particularly those ones used in traditional medicine, are playing an important role in drug discovery [9, 10].

The Apocynaceae family comprising 424 genera and 1500 species are found mainly in tropical and temperate regions [11]. The genus *Aspidosperma* consists of 43 species of tropical distribution, which are found mainly between Mexico and Argentina [12] and is popularly used to treat several diseases [13]. In the Amazon region, the barks of species of the genus *Aspidosperma* are commonly used as infusions in folk medicine against fever and rheumatism due to low toxicity and the absence of contraindications [14]. 

These species are known to be very rich in indole alkaloids like aspidospermine and quebrachamine. Alkaloids are one of the most diverse classes of secondary metabolites found in living organisms, with about 12,000 substances, including indole alkaloids (25% of all the alkaloids). Several anti-inflammatory drugs used in the therapy have the basic chemical structure of nitrogen-containing aromatic rings [15]. 

Even though several studies had been conducted with species of the genus *Aspidosperma* some species are scarcely reported. An example is the species *A. tomentosum*, found in the Brazilian “cerrado,” which is known as “Pau pereiro do campo” or “Peroba do campo.” The most commonly used plant parts are wood for construction, production of household utensils, and musical instruments. Although the genus *Aspidosperma* is known to be rich in indole alkaloids, in this study the flavonoid isorhamnetin, a flavonol aglycone was isolated from *A. tomentosum* and has been reported for its antioxidant activity, cytoprotective capacity, and cardiovascular effects [16]. This study was intended to evaluate the antinociceptive and anti-inflammatory activities of different extracts, fractions, and isorhamnetin of *A. tomentosum* in animal models.

## 2. Materials and Methods

### 2.1. Plant Material

The plant material of *A. tomentosum* was collected in May 2004 in Planaltina, Goiás State, Brazil. The plant material was identified by Dr. J. E. de Paula, from Federal University of Brasília, Brazil, in which a voucher specimen (no. JEP 3732 (UB)) was deposited.

### 2.2. Extract Preparation

**Figure 1:**
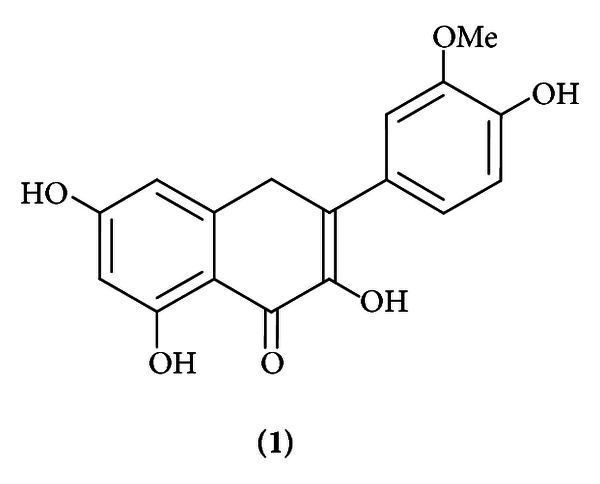
Chemical structure of the flavonoid isorhamnetin (**1**).

The stem bark of the plant was subjected to drying at 40°C for 72 hours with subsequent grinding. The dried and ground material presented total weight of 3.64 kg and was subjected to extraction with 20 L of ethanol 90% in percolator at room temperature for 96 h. Removal of solvent under reduced pressure in rotary device provided 316.55 g (8.696%) of crude concentrate. The crude ethanolic extract (CEE) was subjected to filtration with different organic solvents, with increasing gradient of polarity organic, obtaining the following fractions: Hexane 100% (21.50 g, 6,79%), Hex : CHCl_3_ 50% (103.65 g, 32,74%), CHCl_3_ : EtAcO 50% (88.25 g, 27,88%), CHCl_3_ : MeOH 5% (56.35 g, 17,80%), and CHCl_3_ : MeOH 10% (31.12 g, 9,83%). During the process of concentration of the CEE was noticed the formation of a yellow precipitate (12.56 g, 3,97%). The precipitation was identified using spectral data of UV, IV, ^1^H NMR, and ^13^C NMR as flavonoid 3,5,7,4′-tetrahydroxy-3′-methoxyflavone (isorhamnetin, Figure 1). 

### 2.3. Animals

Male and female Swiss mice weighing 25–30 g with 6–8 weeks age were provided from BIOCEN-UFAL. The animals were housed in groups of five in standard cages at room temperature (27 ± 3°C) in 12 h dark/12 h light control, with both food and water ad libitum. All tests were conducted under the guidelines of the International Association for the Study of Pain [17]. Also, the experiments were authorized by the Ethical Committee for Animal Care of UFAL, Brazil (no. 009468/2006-12).

### 2.4. Drugs and Reagents

Acetic acid, indomethacin, and formaldehyde were purchased from Merck & Co. Arabic gum, dipyrone, Tween 80, and thioglycolate were purchased from Sigma-Aldrich Chemical Co., while morphine, naloxone, and haloperidol were purchased from Cristália-BR. A solution of formalin 2.5% was prepared with formaldehyde in saline. The drugs were used as suspension in Arabic gum in all the experiments and oral administrations.

### 2.5. Acetic Acid-Induced Writhing

The animals were treated with the CEE and its fractions (100 mg/kg, p.o.), as well as isorhamnetin (100 *μ*mol/kg, p.o.), the drug standard dipyrone, (100 *μ*mol/kg, p.o.), and/or vehicle (arabic gum and Tween 80, p.o.). The abdominal contortions were induced by an intraperitoneal injection of a 0.6% acetic acid solution 40 min after the treatment. The number of writhings was counted starting at 5 min after injection of the stimulus during 20 min, and the antinociceptive activity was expressed as the reduction on the number of abdominal writhing [18].

### 2.6. Hot Plate Test

The animals were treated with the CEE and its fractions (100 mg/kg, p.o.), as well as isorhamnetin (100 *μ*mol/kg, p.o.), morphine (15 *μ*mol/kg, p.o.), or vehicle (arabic gum and Tween 80, p.o.). Then, they were placed on a heated surface at 54 ± 1°C, and reaction to the thermal stimulus (lifting or biting its paw) was registered at 30, 60, 90, and 120 min after the treatment. Two measurements were made in intervals of 30 min, establishing the time of “cut-off” of 15 s that were used as a control [19].

### 2.7. Catalepsy

The animals were placed with both forelegs over a horizontal bar glass at a height of 5 cm from the ground. The time that the animal was in that position was clocked up to 300 s, after three attempts. The cataleptic state was considered completed when the animal took off to the bar, or when it went to the bar. The measurement was carried out in 30, 60, 120, 180, and 240 min after administration of CEE and its fractions (100 mg/kg, p.o.) from *A. tomentosum* isorhamnetin (100 *μ*mol/kg, p.o.), the drug standard haloperidol (1 mg/kg), or vehicle [20].

### 2.8. Nociception Induced by Formalin

The animals received a subcutaneous injection of 20 *μ*L of formalin 2.5% into the dorsal right hind paw. Animals were observed from 0 to 5 min (neurogenic phase) and from 15 to 30 min (inflammatory phase), and the time that they spent licking the paw was recorded and considered as indicative of nociception. The CEE, fractions (100 mg/kg), isorhamnetin (100 *μ*mol/kg), indomethacin (100 *μ*mol/kg), and vehicle were administered 40 min before of formalin [21].

### 2.9. Ear Capsaicin-Induced Oedema

This test consists of local administration of 20 *μ*L of a solution of capsaicin diluted in acetone (12.5 mg/mL), 40 min after the administration of CEE, fractions (100 mg/kg), isorhamnetin (100 *μ*mol/kg), and indomethacin (100 *μ*mol/kg). Thirty minutes after capsaicin application, mice were killed and both ears were removed, and after that the weights of the inflamed ears were compared with the weights of the ear against-lateral that was not dealt with by the flogistic agent. Circular sections were taken, using a cork borer with a diameter of 6 mm, and weighed. The increase in weight caused by the irritant was measured by subtracting the weight of the untreated left ear section from that of the treated right ear sections [22].

### 2.10. Thioglycolate-Induced Peritonitis

The mice were treated with the CEE, fractions (100 mg/kg), isorhamnetin (100 *μ*mol/kg), and indomethacin (100 *μ*mol/kg) before administration of 1 mL of thioglycolate 3%. After 4 h, the animals were killed by cervical dislocation. The peritoneal cavity was washed with 3.0 mL of HANKS, and after gentle manual massage the exudate was retrieved and the volume was measured. The exudate was collected and the number of cells was counted in a Neubauer chamber, and the results were expressed as cells × 10^6^/mL. The exudate was collected and used freshly for cell counts and cytospin preparations [23, 24].

### 2.11. Statistical Analysis

Data obtained from animal experiments were expressed as the mean ± standard error of the mean (± SEM). Statistical differences between the treated and the control groups were analyzed statistically by analysis of variance (ANOVA) followed by Dunnett's test, in the tutorial Prisma 3.0. Results with *P < 0.05 and **P < 0.01 were considered significant.

## 3. Results 

### 3.1. Acetic Acid-Induced Abdominal Writhing

**Figure 2:**
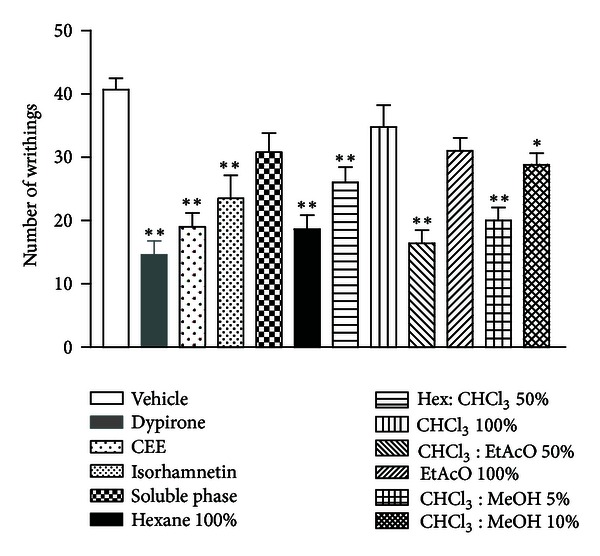
Antinociceptive effect of extract, fractions, and isorhamnetin (100 mg/kg, p.o.) on the acetic acid-induced writhings in mice. Each column represents the mean ± S.E.M. of 6 animals. Statistical differences between the treated and the control groups were evaluated by ANOVA and Dunnett hoc tests, and the asterisks denote the significance levels in comparison with control groups: *P < 0.05; **P < 0.01.

Oral administration of the CEE, isorhamnetin, Hexane 100%, Hex : CHCl_3_ 50%, CHCl_3_ : EtAcO 50%, CHCl_3_ : MeOH 5%, and CHCl_3_ : MeOH 10% produced significant decrease in the number of abdominal writhing response in the acetic acid with 53.3%, 42.2%, 54.7%, 36.1%, 59.7%, 50.8%, and 29.2% of inhibition, respectively (Figure 2). Dipyrone, standard drug used, also produced a significant inhibition of acetic acid-induced abdominal writhing response with 64.1% of inhibition. The animals treated with soluble phase, CHCl_3_ 100%, and EtAcO 100% fractions did not show significant decrease in the number of abdominal writhing.

### 3.2. Hot Plate Test

In the hot plate test, only the animals treated with Hex : CHCl_3_ 50%, CHCl_3_ 100%, and CHCl_3_ : MeOH 5% fractions showed a significant increase of latency time at 30 (only CHCl_3_ : MeOH 5%) and 60 min. Animals treated with morphine showed a significant increase in latency at 30 to 120 min (Table 1). In the presence of naloxone, opioid receptor antagonist, morphine, Hex : CHCl_3_ 50%, CHCl_3_ 100%, and CHCl_3_ : MeOH 5% fractions, its effects were completely blocked (Table 2).

### 3.3. Catalepsy

Fractions that showed significant results in the hot plate test were tested in catalepsy test. In the present study, haloperidol induced a strong cataleptic effect during at the four-hour period of the study. The animals treated with vehicle and fractions Hex : CHCl_3_ 50%, CHCl_3_ 100%, and CHCl_3_ : MeOH 5% did not show catalepsy condition, remaining until 1 min on the bar (Table 3).

### 3.4. Formalin-Induced Nociception

In this test, the treatment with CEE, soluble phase, all fractions, and isorhamnetin caused a significant inhibition of neurogenic phase in the nociception induced by formalin. However, in the inflammatory phase, only CEE, isorhamnetin, CHCl_3_ 100%, and CHCl_3_ : MeOH 5% fractions showed a significant antinociceptive response with 64.8%, 74.2%, 76.2%, and 60.8% of inhibition in the licking induced by the formalin. The treatment with indomethacin was able to inhibit neurogenic and inflammatory phase by 23.4% and 64.8%, respectively (Table 4).

### 3.5. Ear Capsaicin-Induced Oedema

**Figure 3:**
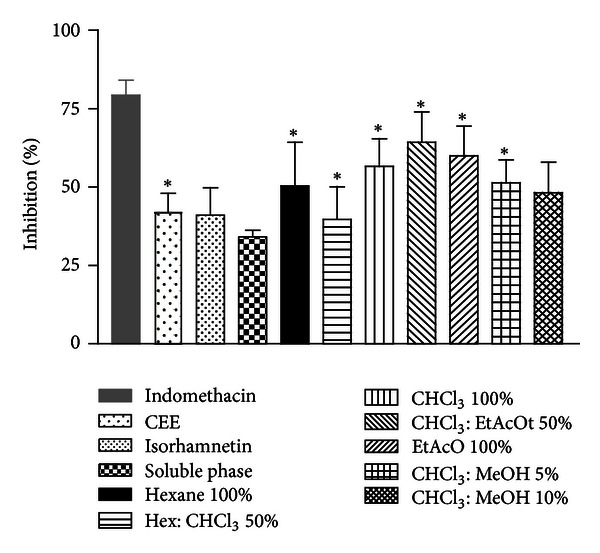
Effects of extract, fractions, and isorhamnetin (100 mg/kg, p.o.) on the ear edema induced by capsaicin model. Each column represents the mean ± S.E.M. of 5 animals. The asterisks denote the significance levels in comparison with control groups, *P < 0.05.

In this test, the CEE and all of fractions, except for CHCl_3_ : MeOH 10% fraction, isorhamnetin, and soluble fraction were able to produce an antioedematogenic activity. Moreover, the CHCl_3_ : EtAcO 50% was the most active with 64.2% of inhibition of edema-induced by capsaicin. Indomethacin also produced a reduction of edema induced by capsaicin by 79.2% (Figure 3).

### 3.6. Thioglycolate-Induced Peritonitis

The ability of CEE, soluble phase, all fractions, and isorhamnetin to inhibit the leukocyte migration was evaluated in the thioglycolate-induced peritonitis. In this test, only EtAcO 100% fraction was not able to significantly inhibit leukocyte migration into the peritoneal cavity (Table 5).

## 4. Discussion 

In this work, we show for the first time that *A. tomentosum* has antinociceptive and anti-inflammatory properties. Such properties were assessed by different animal models. Initially, acetic acid-induced abdominal writhing was carried out. This test is used to evaluate peripheral antinociceptive activities of compounds. Acetic acid injection produces peritoneal inflammation, which triggers a response characterized by abdominal contractions, movements of the body as a whole, twisting of dorsoabdominal muscles, and a reduction in motor activity and motor incoordination [25]. This model involves different nociceptive mechanisms, such as biogenic amines release (e.g., histamine and serotonin), cyclooxygenases, and their metabolites [26]. In this test, our findings showed that the extract, fractions, except for soluble phase, CHCl_3_ 100% and EtAcO 100%, and isorhamnetin induced a significant decrease in the number of abdominal writhing. In addition, these effects can be compared with dipyrone, used as the reference analgesic drug.

The hot plate test, considered a good model for studying central antinociceptive activity, measures the complex response to acute, noninflammatory, nociceptive stimuli and is influenced by opioids. The ability of compounds to prolong latency to discomfort in the hot plate test, as seen with Hex : CHCl_3_ 50%, CHCl_3_ 100%, and CHCl_3_ : MeOH 5% fractions, indicates their ability to influence the central mechanism of pain [27]. Since the hypothesis of a central antinociceptive action was generated, we performed the hot plate test in the presence of an opioid receptor antagonist, naloxone, to investigate a possible mechanism of action via opioid receptors. The results show that in presence of the opioid antagonist, the effect was abolished, suggesting that these fractions have constituents which may be acting by a mechanism of action dependent on opioid receptors. This central antinociceptive activity of these fractions might be due to constituents as alkaloids. *The Aspidosperma* genus is known to be very rich in indole alkaloids [15]. It has been suggested that the central antinociceptive action of indole alkaloid, such as mesaconitine, lappaconitine, and 3-acetylaconitine, is linked to the noradrenergic and serotoninergic systems [28]. To investigate if the fractions Hex : CHCl_3_ 50%, CHCl_3_ 100%, and CHCl_3_ : MeOH 5% from *A. tomentosum* also act on adrenergic and serotoninergic systems or acetylcholine and dopamine receptors, the catalepsy test was performed. 

Numerous experimental data have indicated that administration of typical neuroleptics like haloperidol induce catalepsy in rats, a phenomenon generally defined as the long-term maintenance of the animal in an abnormal posture [29]. Haloperidol is thought to induce catalepsy through the blockade of dopamine receptors in the striatum [30] and nucleus accumbens [31]. Along with the blockade of postsynaptic striatal dopamine receptors, multiple other mechanisms have been proposed to explain the catalepsy behavior such as antagonism of *μ*-opioid or acetylcholine receptors [32, 33]. Furthermore, the catalepsy state may be modulated by drugs that modify the serotonergic or purinergic neurotransmitter systems [34, 35]. In the catalepsy test, the fractions did not induce a cataleptic condition. These results suggest that they are not acting through these mechanisms of action.

The neurogenic and inflammatory pain was evaluated using nociception induced by formalin. It is known that neurogenic phase (first phase) has been associated with direct effect of formalin on nociceptors, while inflammatory phase (second phase) is said to involve inflammatory response [36–38]. Different mechanisms have been shown to be involved in neurogenic and inflammatory phases of nociceptive inflammation, based on the different pharmacological mechanisms of action. For example, while second phase behaviors are selectively attenuated by cyclooxygenase inhibitors, neurogenic and inflammatory phase behaviors are attenuated by opioids [37]. Because better performances of the crude ethanolic extract, fractions, and isorhamnetin on the neurogenic phase of formalin could be observed, we might suggest a neurogenic antinociceptive action of the *A. tomentosum*. Moreover, treatment with the CEE, CHCl_3_ 100% fraction, CHCl_3_ : EtAcO 50% fraction, CHCl_3_ : MeOH 5% fraction, and isorhamnetin protected the inflammatory phase too, indicating a possible anti-inflammatory activity.

Based on these data, this study strongly supports that systemic administration of CEE, soluble phase, fractions, and isorhamnetin from *A. tomentosum* modulates peripheral and central nociceptive response. A possible inhibitory effect of *A. tomentosum* in cells recruitment into the peritoneal cavity was evaluated by thioglycolate-induced peritonitis. The local injection of thioglycolate 3% caused extravasation of polymorphonuclear leukocytes into the peritoneal cavity, peaking at 4 h and still elevate above basal levels at 48 h after injection [39]. Leukotriene B4 and C5a, potent chemotactic molecules that accumulate early in inflamed tissue, drive thioglycollate-induced peritonitis, and at least the leukotriene component is, in turn, regulated by reactive oxygen species (ROS) [40]. This may explain the activity showed by isorhamnetin, a flavonoid which is a potential free-radical scavenger and also attenuated LOX-1 upregulation. Furthermore, the number of cells in the peritoneal fluids was reduced after administration of the CEE, soluble phase, and all fractions, except for EtAcO 100% fraction, from *A. tomentosum* in regard with their anti-inflammatory actions.

In order to analyze the effects of CEE, soluble phase, fractions, and isorhamnetin from *A. tomentosum* in other components of the inflammatory response, these ones were studied using capsaicin-induced ear edema in mice. In this model, topical application of capsaicin, the main pungent ingredient in “hot” chili peppers, when applied to the ear of mice, produces neurogenic acute inflammatory responses, such as axon reflex vasodilatation, plasma leakage, and erythema [41]. Capsaicin and related vanilloid compounds produce burning pain by depolarizing specific subsets of C and A*δ* nociceptors through activation of the vanilloid receptor, TRPV1 [42]. The isorhamnetin, soluble phase, and fraction CHCl_3_ : MeOH 10% did not present a significant inhibition of percentage of oedema, suggesting that its action is not related with vanilloid receptor and mediators involved after capsaicin action. On the other hand, as the CEE and the other fractions showed activity in this model, it suggests that they contain compounds which may antagonize capsaicin actions.

There are studies showing that isorhamnetin reduces inducible nitric-oxide synthase (iNOS) expression, and this effect may well be mediated by inhibition of NF-*κ*B activation. Because NF-*κ*B is involved in the activation of several inflammatory genes, flavonoids that inhibit activation of NF-*κ*B are likely to downregulate production of an array of inflammatory mediators in addition to iNOS [43, 44]. The isorhamnetin is a potential free-radical scavenger and showed appreciable effects against 1,1-diphenyl-2-picrylhydrazyl (DPPH) radical-generating system. 

Taken together, the results showed herein suggest that *A. tomentosum* has antinociceptive and anti-inflammatory activities. Furthermore, Hex : CHCl_3_ 50%, CHCl_3_ 100%, and CHCl_3_ : MeOH 5% fractions have compounds with central antinociceptive activity which may act through opiate receptors, and the anti-inflammatory activity showed by isorhamnetin probably involves its antioxidant activity by free radical scavenging.

## 5. Conclusion 

In conclusion, this study has shown that the CEE, the fractions, and isorhamnetin from *A. tomentosum* have significant antinociceptive and anti-inflammatory effects in mice at the doses and routes investigated. However, pharmacological and chemical studies are needed in order to characterize the mechanism(s) responsible for the antinociceptive and anti-inflammatory actions and also to identify other active agents present in this plant.

## Figures and Tables

**Table 1:** Effect of extract, fractions, and isorhamnetin in the hot plate test.

Group	Pretreatment^†^	Posttreatment^†^			
	0 min	30 min	60 min	90 min	120 min
Vehicle	4.2 ± 0.7	3.7 ± 0.7	3.7 ± 0.6	3.7 ± 0.7	4.7 ± 1.2
Morphine	12.8 ± 0.4**	10.3 ± 0.8**	9.7 ± 0.7**	9.7 ± 0.9**	12.8 ± 0.4**
CEE	4.3 ± 0.5	4.8 ± 1.8	6.6 ± 1.2	5.9 ± 1.4	6.3 ± 0.9
Isorhamnetin	4.1 ± 0.5	4.7 ± 0.8	4.4 ± 0.7	3.6 ± 0.8	7.2 ± 1.7
Soluble phase	4.6 ± 0.7	5.0 ± 0.7	4.6 ± 0.8	3.1 ± 0.4	2.9 ± 0.5
Hexane 100%	3.2 ± 0.7	2.6 ± 0.4	3.0 ± 0.4	2.8 ± 0.5	3.1 ± 0.6
Hex : CHCl_3_ 50%	4.6 ± 0.3	6.3 ± 0.9	7.4 ± 0.7*	7.0 ± 1.5	7.3 ± 1.3
CHCl_3_ 100%	3.0 ± 0.6	4.8 ± 0.6	7.8 ± 0.9*	5.3 ± 0.2	6.3 ± 1.5
CHCl_3_ : EtAcO 50%	3.9 ± 0.7	4.3 ± 0.7	3.6 ± 0.4	3.3 ± 0.8	3.9 ± 0.8
EtAcO 100%	4.3 ± 1.0	2.9 ± 0.5	4.2 ± 0.6	5.4 ± 1.4	3.7 ± 0.6
CHCl_3_ : MeOH 5%	3.2 ± 0.8	8.5 ± 1.2**	7.7 ± 1.6*	6.7 ± 1.7	7.2 ± 1.1
CHCl_3_ : MeOH 10%	4.4 ± 0.8	5.2 ± 1.2	6.7 ± 0.7	6.9 ± 1.1	6.6 ± 1.2

^†^Data represented as mean ± S.E.M. Number of animals = 8. *P < 0.05; **P < 0.01. (One-way ANOVA and Dunnett test.)

**Table 2:** Effect of fractions Hex : CHCl_3_ 50%, CHCl_3_ 100%, and CHCl_3_ : MeOH 5% in the hot plate test, in the presence of the drug nalaxone.

Group	Pretreatment^†^	Posttreatment^†^			
	0 min	30 min	60 min	90 min	120 min
Vehicle	4.2 ± 0.8	3.7 ± 0.7	3.7 ± 0.6	3.7 ± 0.7	4.7 ± 1.2
Morphine	5.9 ± 0.3	12.8 ± 0.4**	10.3 ± 0.8**	9.7 ± 0.7**	9.7 ± 0.95**
Morphine + Nlx	2.6 ± 0.5	3.3 ± 0.9	3.8 ± 0.7	2.6 ± 0.8	3.0 ± 0.4
Hex : CHCl_3_ 50% + Nlx	2.6 ± 0.3	3.7 ± 0.5	1.2 ± 0.2	3.6 ± 0.8	2.4 ± 0.4
CHCl_3_ 100% + Nlx	2.6 ± 0.4	4.2 ± 0.5	2.0 ± 0.4	3.2 ± 0.6	2.7 ± 0.3
CHCl_3_ : MeOH 5% + Nlx	3.2 ± 0.2	3.9 ± 0.8	3.0 ± 0.7	3.3 ± 0.5	5.0 ± 1.2

^†^Data represented as mean ± S.E.M. Number of animals = 8. *P < 0.05; **P < 0.01. (One-way ANOVA and Dunnett test.)

**Table 3:** Effect of fractions Hex : CHCl_3_ 50%, CHCl_3_ 100%, and CHCl_3_ : MeOH 5% on catalepsy test.

Group	30 min	60 min	120 min	180 min	240 min
Vehicle	5.0 ± 0.6	3.7 ± 1.1	6.5 ± 2.5	8.7 ± 2.9	5.7 ± 0.8
Haloperidol	103.2 ± 17.1***	174.8 ± 37.3***	201.6 ± 36.6***	230.5 ± 44.0***	261.8 ± 28.5***
Hex : CHCl_3_ 50%	10.7 ± 2.0	22.1 ± 9.4	20.8 ± 7.4	19.5 ± 6.3	11.9 ± 2.9
CHCl_3_ 100%	6.3 ± 1.9	4.3 ± 0.6	10.9 ± 2.5	13.0 ± 4.3	5.8 ± 0.7
CHCl_3_ : MeOH 5%	6.9 ± 2.9	7.9 ± 2.0	11.1 ± 5.1	10.9 ± 3.4	22.0 ± 7.9

^†^Data represented as mean ± S.E.M, number of animals = 6. ***P < 0.001 (One-way ANOVA and Dunnett test.)

**Table 4:** Effects of extract, fractions (100 mg/kg, p.o.), isorhamnetin, and indomethacin (100 *μ*mol/kg, p.o.) in nociception induced by formalin.

Group	*n*	Linking (s)		% of inhibition	
		Phase 1	Phase 2	Phase 1	Phase 2
		Mean ± S.E.M.	Mean ± S.E.M.	Mean ± S.E.M.	Mean ± S.E.M.
Vehicle	6	82.8 ± 5.6	215.7 ± 24.0	—	—
Indomethacin	6	59.4 ± 8.1	94.6 ± 24.4	23.4*	56.1**
CEE	6	39.6 ± 2.4	75.8 ± 29.7	55.0**	64.8**
Isorhamnetin	6	59.4 ± 6.3	55.6 ± 32.7	35.7**	74.2**
Soluble phase	6	41.8 ± 3.4	139.6 ± 10.5	52.3**	35.3
Hexane 100%	6	59.2 ± 4.4	195.0 ± 28.2	31.8**	9.6
Hex : CHCl_3_ 50%	6	62.7 ± 3.9	204.5 ± 42.4	24.2**	5.2
CHCl_3_ 100%	6	35.5 ± 7.0	51.2 ± 19.3	57.1**	76.2**
CHCl_3_ : EtAcO 50%	6	46.0 ± 5.2	131.2 ± 18.4	49.6**	39.2
EtAcO 100%	6	56.0 ± 2.8	128.8 ± 9.7	32.4**	40.3
CHCl_3_ : MeOH 5%	6	59.6 ± 3.7	84.6 ± 27.7	31.8**	60.8**
CHCl_3_ : MeOH 10%	6	42.2 ± 2.6	177.8 ± 16.3	52.0**	17.6

Statistical differences between the treated and the control groups were evaluated by ANOVA and Dunnett test, and the asterisks denote the significance levels in comparison with control groups: *P < 0.05; **P < 0.01.

**Table 5:** Effects of extract, fractions (100 mg/kg, p.o.), and isorhamnetin (100 *μ*mol/kg, p.o.) in the thioglycolate-induced peritonitis.

Group	*n*	Cell number × 10^6^/mL	% of inhibition
		Mean ± S.E.M.	Mean ± S.E.M.
Vehicle	6	1.5 ± 1.0	—
CEE	6	3.6 ± 0.4	57.6%**
Isorhamnetin	6	4.6 ± 0.6	45.9%**
Soluble phase	6	2.2 ± 0.3	74.1%**
Hexane 100%	6	3.4 ± 0.4	60.0%**
Hex : CHCl_3_ 50%	6	1.7 ± 0.2	80.0%**
CHCl_3_ 100%	6	3.8 ± 0.5	55.3%**
CHCl_3_ : EtAcO 50%	6	3.2 ± 0.6	62.3%**
EtAcO 100%	6	8.6 ± 1.8	0.0%
CHCl_3_ : MeOH 5%	6	2.4 ± 0.5	71.8%**
CHCl_3_ : MeOH 10%	6	4.6 ± 0.5	45.9%**

Statistical differences between the treated and the control groups were evaluated by ANOVA and Dunnett tests, and the asterisks denote the significance levels in comparison with control groups, **P < 0.01.

## References

[1] Levine JD, Reichling DB, Wall PD, Melzack R (1999). Peripheral mechanisms of inflammatory pain. *Textbook of Pain*.

[2] Basbaum AI, Bautista DM, Scherrer G, Julius D (2009). Cellular and molecular mechanisms of pain. *Cell*.

[3] Medzhitov R (2010). Inflammation 2010: new adventures of an old flame. *Cell*.

[4] Hua S, Cabot PJ (2010). Mechanisms of peripheral immunecell-mediated analgesia in inflammation: clinical and therapeutic implications. *Cell*.

[5] Gris P, Gauthier J, Cheng P (2010). A novel alternatively spliced isoform of the mu-opioid receptor: functional antagonism. *Molecular Pain*.

[6] Ernst E (2002). Adulteration of Chinese herbal medicines with synthetic drugs: a systematic review. *Journal of Internal Medicine*.

[7] Fennerty MB (2001). NSAID-related gastrointestinal injury: evidence-based approach to a preventable complication. *Postgraduate Medicine*.

[8] Singh G (1998). Recent considerations in nonsteroidal anti-inflammatory drug gastropathy. *American Journal of Medicine*.

[9] Newman DJ, Cragg GM (2007). Natural products as sources of new drugs over the last 25 years. *Journal of Natural Products*.

[10] Abraham DJ, Hoboken W, Buss AD, Cox B, Waigh RD (2003). *Burger’s Medicinal Chemistry and Drug Discovery*.

[11] Endress ME, Bruyns PV (2000). A revised classification of the apocynaceae s.l.. *Botanical Review*.

[12] Marcondes-Ferreira W, Kinoshita LS (1996). Uma nova divisão infragenérica para *Aspidosperma* Mart. (Apocinaceae). *Revista Brasileira de Botânica*.

[13] Pereira MM (2007). Alcalóides indólicos isolados de espécies do gênero *Aspidosperma* (APOCYNACEAE). *Química Nova*.

[14] Schultes RE, Raffauf RF (1990). *The Healing Forest: Medicinal and Toxic Plants of the Northwest Amazonia*.

[15] Deutsch HF, Evenson MA, Drescher P, Sparwasser C, Madsen PO (1994). Isolation and biological activity of aspidospermine and quebrachamine from an *Aspidosperma* tree source. *Journal of Pharmaceutical and Biomedical Analysis*.

[16] Teng BS, Lu YH, Wang ZT, Tao XY, Wei DZ (2006). In vitro anti-tumor activity of isorhamnetin isolated from *Hippophae rhamnoides* L. against BEL-7402 cells. *Pharmacological Research*.

[17] Zimmermann M (1983). Ethical guidelines for investigations of experimental pain in conscious animals. *Pain*.

[18] Collier HO, Dinneen LC, Johnson CA, Schneider C (1968). The abdominal constriction response and its suppression by analgesic drugs in the mouse.. *British Journal of Pharmacology*.

[19] Kuraishi Y, Harada Y, Aratani S (1983). Separate involvement of the spinal noradrenergic and serotonergic systems in morphine analgesia: the differences in mechanical and thermal analgesic tests. *Brain Research*.

[20] Sanberg PR, Bunsey MD, Giordano M, Norman AB (1988). The catalepsy test: its ups and downs. *Behavioral Neuroscience*.

[21] Hunskaar S, Hole K (1987). The formalin test in mice: dissociation between inflammatory and non-inflammatory pain. *Pain*.

[22] Sánchez T, Moreno JJ (1999). Role of prostaglandin H synthase isoforms in murine ear edema induced by phorbol ester application on skin. *Prostaglandins and Other Lipid Mediators*.

[23] Desouza IA, Ribeiro-DaSilva G (1998). Neutrophil migration induced by staphylococcal enterotoxin type A in mice: a pharmacological analysis. *European Journal of Pharmacology*.

[24] Smith SR, Denhardt G, Terminelli C (2001). The anti-inflammatory activities of cannabinoid receptor ligands in mouse peritonitis models. *European Journal of Pharmacology*.

[25] Le Bars D (2001). Animal models of nociception. *Pharmacological Reviews*.

[26] Duarte IDG, Nakamura M, Ferreira SH (1988). Participation of the sympathetic system in acetic acid-induced writhing in mice. *Brazilian Journal of Medical and Biological Research*.

[27] Pini LA, Vitale G, Ottani A, Sandrini M (1997). Naloxone-reversible antinociception by paracetamol in the rat. *Journal of Pharmacology and Experimental Therapeutics*.

[28] Ameri A (1998). The effects of Aconitum alkaloids on the central nervous system. *Progress in Neurobiology*.

[29] Costall B, Fortune DH, Naylor RJ (1975). Serotonergic involvement with neuroleptic catalepsy. *Neuropharmacology*.

[30] Ellenbroek B, Schwarz M, Sontag KH (1985). Muscular rigidity and delineation of a dopamine-specific neostriatal subregion: tonic EMG activity in rats. *Brain Research*.

[31] Hartgraves SL, Randall PK (1986). Dopamine agonist-induced stereotypic grooming and self-mutilation following striatal dopamine depletion. *Psychopharmacology*.

[32] Havemman U, Kuschinsky K (1981). Further characterization of ipiod receptors in the striatum mediating muscular rigidity in rats. *Naunyn- Schmiedeberg’s Archives of Pharmacology*.

[33] Moo-Puc RE, Góngora-Alfaro JL, Alvarez-Cervera FJ, Pineda JC, Arankowsky-Sandoval G, Heredia-López F (2003). Caffeine and muscarinic antagonists act in synergy to inhibit haloperidol-induced catalepsy. *Neuropharmacology*.

[34] Pires JGP, Ramage AG, Silva SR, Futuro-Neto HA (1993). Effects of the 5-HT receptor antagonists cyanopindolol, ICI 169,369, cisapride and granisetron on neuroleptic-induced catalepsy in mice. *Brazilian Journal of Medical and Biological Research*.

[35] Zarrindast MR, Iraie F, Heidari MR, Mohagheghi-Badi M (1997). Effect of adenosine receptor agonists and antagonists on morphine-induced catalepsy in mice. *European Journal of Pharmacology*.

[36] Dickenson AH, Sullivan AF (1987). Peripheral origins and central modulation of subcutaneous formalin-induced activity of rat dorsal horn neurones. *Neuroscience Letters*.

[37] Yaksh TL, Ozaki G, McCumber D (2001). An automated flinch detecting system for use in the formalin nociceptive bioassay. *Journal of Applied Physiology*.

[38] Shibata M, Ohkubo T, Takahashi H, Inoki R (1989). Modified formalin test: characteristic biphasic pain response. *Pain*.

[39] Ajuebor MN, Das AM, Virág L, Flower RJ, Szabó C, Perretti M (1999). Role of resident peritoneal macrophages and mast cells in chemokine production and neutrophil migration in acute inflammation: evidence for an inhibitory loop involving endogenous IL-10. *Journal of Immunology*.

[40] Segal BH, Kuhns DB, Ding L, Gallin JI, Holland SM (2002). Thioglycollate peritonitis in mice lacking C5, 5-lipoxygenase, or p47phox: complement, leukotrienes, and reactive oxidants in acute inflammation. *Journal of Leukocyte Biology*.

[41] Inoue H, Nagata N, Koshihara Y (1993). Profile of capsaicin-induced mouse ear oedema as neurogenic inflammatory model: comparison with arachidonic acid-induced ear oedema. *British Journal of Pharmacology*.

[42] Caterina MJ, Schumacher MA, Tominaga M, Rosen TA, Levine JD, Julius D (1997). The capsaicin receptor: a heat-activated ion channel in the pain pathway. *Nature*.

[43] Bao M, Lou Y (2006). Isorhamnetin prevent endothelial cell injuries from oxidized LDL via activation of p38MAPK. *European Journal of Pharmacology*.

[44] Hämäläinen M, Nieminen R, Vuorela P, Heinonen M, Moilanen E (2007). Anti-inflammatory effects of flavonoids: genistein, kaempferol, quercetin, and daidzein inhibit STAT-1 and NF-*κ*B activations, whereas flavone, isorhamnetin, naringenin, and pelargonidin inhibit only NF-*κ*B activation along with their inhibitory effect on iNOS expression and NO production in activated macrophages. *Mediators of Inflammation*.

